# Improving vaccination coverage among 24-month-old children in the ASL Napoli 3 Sud: a M.A.S.T.E.R. approach to transformation, evaluation and strategic redesign

**DOI:** 10.3389/fpubh.2026.1901337

**Published:** 2026-08-10

**Authors:** Arianna Scala, Sabrina Cristillo, Maria Giuliana Del Piano, Teresa Di Lorenzo, Anna Rita Maestro, Claudia Esposito, Pasqualina Mascolo, Sara Avagliano, Francesco Girardi, Pasquale Izzo, Giuseppina Iannone, Eliana Raiola, Giovanni Improta

**Affiliations:** 1 Department of Public Health, University of Naples Federico II, Naples, Italy; 2 Local Health Authority, Napoli 3 Sud, Torre del Greco, Italy

**Keywords:** continuous improvement, lean management, M.A.S.T.E.R. methodology, P01C indicator, pediatric vaccinations, public health, vaccination coverage, vaccination monitoring

## Abstract

**Background:**

Pediatric vaccination coverage is a key indicator of primary prevention and collective protection. Indicator P01C of the New Guarantee System measures coverage among 24-month-old children for the complete primary series and is nationally monitored within the Essential Levels of Care for collective prevention. In ASL Napoli 3 Sud, P01C coverage decreased from 94.57% in 2022 to 92.75% in 2023, revealing organizational, informational, and operational weaknesses.

**Objective:**

This study describes the use of M.A.S.T.E.R. (Methodological Approach for Strategic Transformation, Evaluation, and Redesign) as an organizational framework to analyze the decline in P01C coverage, coordinate a multi-component improvement programme, and support monitoring.

**Methods:**

This before-and-after quality improvement project was conducted as an implementation-oriented evaluation within a pediatric vaccination service. The period was 2022–2024: 2022 was the historical reference, 2023 the pre-intervention year, and 2024 the implementation/post-intervention assessment year. M.A.S.T.E.R. is a pre-existing QI framework combining Lean Management, DMAIC, PDCA/PDSA, and A3 thinking. The programme included standardized recalls, digital calendars, dashboards, staff training, data cleansing and integration, logistics review, temporary staffing support, and collaboration with Primary Care Pediatricians. Annual P01C coverage was summarized descriptively using aggregate percentages and absolute/relative changes.

**Results:**

Critical issues concerned active outreach, cohort tracking, data integration, Sinfonia updates, pediatrician coordination, vaccine logistics, and district variability. In 2024, P01C coverage returned to 94.57%, matching 2022 but remaining below national benchmark of >95%. Change from 2023 was +1.82 percentage points (+1.96%), indicating restoration of previous performance rather than improvement beyond historical level. Recalls increased from 1,248 to 2,136; rescheduled and completed appointments from 286 to 612; late Sinfonia registrations decreased from 21.4 to 8.6%; pediatrician adherence increased from 54.2 to 87.3%; and vaccine distribution time decreased from 7.4 to 3.1 days. Improved recording completeness and timeliness may have contributed to these differences.

**Conclusion:**

A multi-component programme organized through M.A.S.T.E.R. was associated with redesign of the pediatric vaccination process and restoration of P01C coverage to the 2022 level. Further evaluation is needed to assess causality, transferability, and long-term sustainability. Transferability requires further evaluation, contextual adaptation, and prospective monitoring.

## 1 Introduction

Vaccination is one of the main tools for primary prevention in public health, contributing substantially to the reduction of morbidity and mortality associated with infectious diseases. Recent estimates suggest that vaccination has prevented millions of deaths and has made a major contribution to improving global child survival (1). However, the effectiveness of vaccination programmes depends not only on vaccine availability, but also on maintaining high coverage rates in the target population and on the ability of health services to reach all eligible individuals in a timely and equitable manner. Failure to achieve adequate coverage can lead to the persistence or resurgence of vaccine-preventable diseases, and the literature has shown that even limited declines in routine coverage may be associated with outbreaks in previously controlled areas (2). Globally, coverage rates for several routine childhood vaccines remain below recommended targets, highlighting the need to strengthen immunization systems, improve data quality, and reduce inequalities in access to preventive care (3). In this context, continuous monitoring of vaccination coverage, timely identification of critical areas, and the adoption of targeted organizational strategies are essential to ensuring the sustainability of vaccination programmes, preventing declines in performance, and supporting collective protection. An effective vaccination system encompasses the organizations, institutions, resources, processes, and activities involved in vaccine delivery, including identification of the target population, appointment scheduling, procurement and inventory management, vaccine administration by trained personnel, timely recording of administered doses, and continuous monitoring of coverage (4). The synchronization of these components is a necessary condition to ensure that every eligible individual has the opportunity to be vaccinated correctly and on schedule (5). The quality of vaccination data is generally defined by the dimensions of accuracy, completeness, and timeliness; shortcomings in these areas may compromise the ability to monitor coverage and guide policy decisions (6, 7). The Campania Region has implemented the Sinfonia platform for monitoring and recording vaccinations, providing a digital infrastructure to support the collection and analysis of vaccination data (8). However, the management of vaccination programmes remains complex. Electronic vaccination registries, while enabling rapid data collection and analysis, may face limitations in linking data across different providers, thereby reducing the completeness of coverage estimates (9). Active reminder and recall strategies have been shown to improve vaccination adherence (10), and combined postal and telephone reminders have been reported as effective in pediatric populations (11). The involvement of Primary Care Pediatricians is another key factor, because they are often the main healthcare professionals advising families on vaccination and represent an important source of vaccine-related information (12). Effective coordination among Pediatric Vaccination Centers, organizational units, Primary Care Pediatricians, information systems and pharmaceutical logistics is therefore essential to ensure timely vaccination, up-to-date records, efficient vaccine distribution, and reliable monitoring of performance indicators (13–15). In this context, Lean Management has been widely used to improve healthcare processes, reduce waste, and enhance service quality. It includes tools such as Define, Measure, Analyze, Improve, Control (DMAIC), Value Stream Mapping, Suppliers, Inputs, Process, Outputs, Customers (SIPOC), Ishikawa diagrams, and 5S (16). Frameworks such as DMAIC and Plan-Do-Check-Act/Plan-Do-Study-Act (PDCA/PDSA) offer structured methods for analyzing, implementing, and monitoring healthcare processes (17). A3 thinking also supports structured problem solving, visual synthesis, team alignment, and transparent communication of quality improvement activities (18). However, complex public health processes often require integrated and adaptive approaches. From this perspective, quality can be viewed as an emergent property consolidated through iterative cycles of improvement, audits, and feedback aimed at clear corrective actions (19). In this study, M.A.S.T.E.R. (Methodological Approach for Strategic Transformation, Evaluation, and Redesign) was used as a structured quality improvement framework rather than as a new measurement instrument. M.A.S.T.E.R. is a pre-existing methodological framework and was not developed specifically for the present vaccination project. It organizes established quality improvement tools into six operational phases: Mapping & Contextualization, Analysis & Diagnosis, Strategic Design, Testing & Adaptive Execution, Embedding & Sustainability, and Resilience & Renewal. Previous applications in healthcare settings have described its use for organizing process mapping, root cause analysis, standardization, dashboards, indicators, audits, and control cycles (20–23). These applications support its practical feasibility and contextual transferability, but should not be interpreted as formal validation of M.A.S.T.E.R. as a universal or standardized measurement instrument.

In the present study, the framework was applied as an organizing structure to guide the analysis of the P01C decline, the design of corrective actions, their implementation, and the consolidation of monitoring procedures. The novelty of M.A.S.T.E.R. does not lie in replacing established quality improvement approaches, but in organizing them into a broader implementation pathway that explicitly links diagnosis, prioritization, adaptive execution, routine embedding, sustainability, and resilience. Unlike traditional PDCA/PDSA cycles, which mainly focus on iterative testing and refinement, M.A.S.T.E.R. also includes structured diagnosis, prioritization, routine embedding, sustainability planning, and resilience-oriented renewal. Compared with Lean Six Sigma, which is primarily structured around waste reduction, variation control, and process optimization, M.A.S.T.E.R. places greater emphasis on translating indicator deviations into an end-to-end organizational governance pathway. The selection of indicator P01C was based on both institutional and public health considerations. P01C is part of the New Guarantee System, which is used in Italy to monitor the provision of Essential Levels of Care. Within this system, P01C belongs to the area of collective prevention and public health and specifically assesses vaccination coverage among 24-month-old children for the complete primary series. Its relevance is not limited to final coverage measurement: the indicator reflects the ability of the vaccination system to identify the eligible birth cohort, ensure timely access to scheduled vaccinations, maintain reliable information flows, and guarantee homogeneous service delivery across territories. According to the technical specifications of the New Guarantee System, the threshold value for P01C is greater than 95% (24). For these reasons, P01C was considered a strategic and nationally monitored quality indicator, suitable for evaluating the performance of pediatric vaccination governance and for guiding organizational improvement actions. The objective of this study is to describe the use of the M.A.S.T.E.R. methodology as an organizational framework for addressing the decline observed in the P01C vaccination coverage indicator in ASL Napoli 3 Sud, coordinating a multi-component improvement programme, and monitoring the observed post-implementation recovery in 2024.

## 2 Materials and methods

### 2.1 Study design and organisational context

The study was not designed as a randomized, controlled, or individual-level effectiveness study. The study focused on the pediatric vaccination process related to indicator P01C of the New Guarantee System, which measures vaccination coverage among 24-month-old children for the complete primary vaccination series. Indicator P01C was selected following the decline observed in 2023, when vaccination coverage fell from 94.57% in 2022 to 92.75%, moving further away from the national benchmark of greater than 95%. This decline signaled organizational, informational, and operational challenges relevant to maintaining high pediatric vaccination coverage and achieving national and regional prevention goals.

P01C was also selected because it represents a strategic, nationally monitored measure of pediatric vaccination performance within the New Guarantee System. The indicator is linked to the monitoring of Essential Levels of Care in the area of collective prevention and public health and measures the proportion of children vaccinated by 24 months of age with the complete primary series. Its prioritization was therefore not based only on the observed decline, but also on its institutional relevance, its role in assessing coverage, accessibility, and equity, and its capacity to reflect multiple organizational components of the vaccination process, including cohort identification, appointment management, active recall, vaccine administration, data recording, information-system timeliness, and territorial coordination. For these reasons, P01C was considered an appropriate tracer indicator for applying a structured QI pathway based on the M.A.S.T.E.R. methodology.

The study period covered three calendar years, from 2022 to 2024. The year 2022 was retained as a historical reference period, representing the coverage level before the observed decline. The year 2023 was considered the pre-intervention baseline year, during which the decrease in P01C vaccination coverage was identified and analyzed. The year 2024 represented the implementation and post-intervention assessment year, during which corrective actions were progressively introduced, monitored, and evaluated. The operational implementation of the improvement plan lasted one calendar year, with progressive rollout across the districts of ASL Napoli 3 Sud during 2024. Accordingly, the before-and-after comparison used 2023 as the pre-intervention period and 2024 as the post-intervention period, while the 2022 value was used to verify whether coverage returned to the level observed before the decline. The expected improvement target was therefore defined as restoration of the pre-decline performance level, rather than achievement of coverage beyond the historical 2022 value.

The primary outcome was the annual P01C vaccination coverage value. Intermediate process indicators were used to describe the organizational mechanisms accompanying the observed change, including active outreach, appointment recovery, timeliness of registration in Sinfonia, adherence of Primary Care Pediatricians to shared protocols, vaccine logistics, and inter-district variability.

The study adopted a process improvement approach based on the M.A.S.T.E.R. methodology, which was used as a structured framework to analyze the problem, identify organizational causes, design corrective actions, monitor their implementation, and consolidate the results achieved. M.A.S.T.E.R. was selected because it provides a structured sequence for translating a performance deviation into an improvement pathway. In this study, it was used as an operational, organizational, and implementation framework, not as a validated measurement scale, an isolated intervention, or a stand-alone determinant of vaccination coverage. The framework was not developed specifically for this study; rather, it was adapted to the pediatric vaccination process of ASL Napoli 3 Sud. Its previous healthcare applications were considered useful to support feasibility and methodological consistency, while the present study represents a context-specific application to a nationally monitored pediatric vaccination indicator.

**Figure 1:**
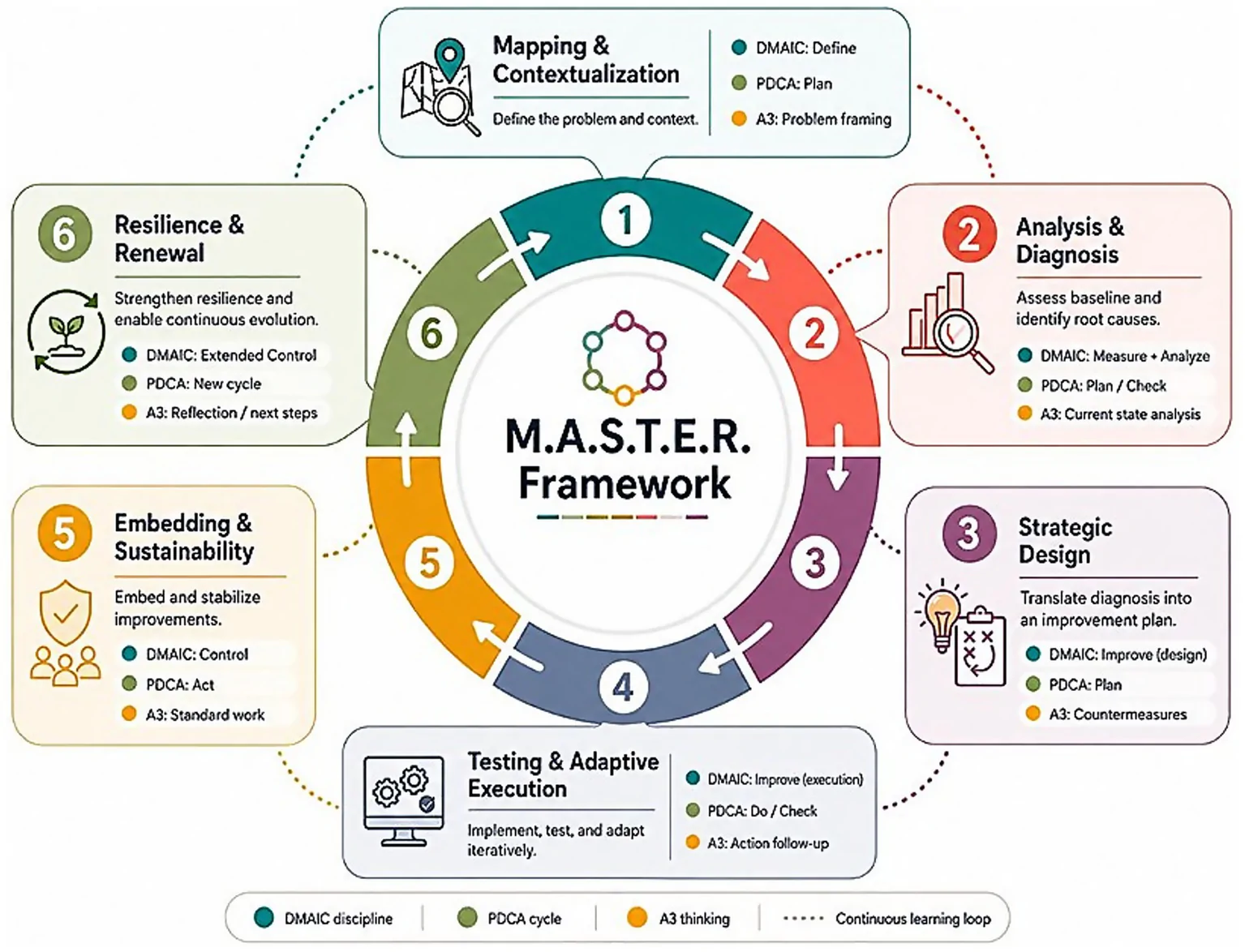
M.A.S.T.E.R. framework for the continuous improvement of pediatric vaccination processes.

The M.A.S.T.E.R. methodology integrates principles of Lean Management, the Define, Measure, Analyze, Improve, Control (DMAIC) cycle, Plan-Do-Check-Act/Plan-Do-Study-Act (PDCA/PDSA), and A3 thinking. Together, these components form a structured process that guides problem analysis, intervention design, implementation, monitoring, and organizational consolidation. As shown in Figure 1, the framework was applied according to six sequential and iterative phases: Mapping & Contextualization, Analysis & Diagnosis, Strategic Design, Testing & Adaptive Execution, Embedding & Sustainability, and Resilience & Renewal.

In this study, the M.A.S.T.E.R. framework was used to structure, coordinate, and monitor a set of concurrent improvement activities. These activities included active recall standardization, digital scheduling, dashboard-based monitoring, data integration, staff training, logistics redesign, temporary staffing reinforcement, and strengthened collaboration with Primary Care Pediatricians. Because these components were implemented within the same improvement pathway, the study was not designed to isolate the independent effect of each component or of the framework itself.

Each phase was adapted to the organizational context of ASL Napoli 3 Sud and involved the main operational units engaged in the pediatric vaccination process: the Epidemiology and Prevention Unit, Pediatric Vaccination Centers, the Maternal and Child Health Unit, the Primary Care Unit, Primary Care Pediatricians, the Pharmaceutical Unit, the Information Systems Unit, the Health Directorate, and the Administrative Directorate. The Epidemiology and Prevention Unit coordinated epidemiological surveillance, P01C monitoring, data analysis, and indicator reporting. Pediatric Vaccination Centers managed appointments, active recalls, vaccine administration, follow-up of missed or delayed vaccinations, and timely recording of administered doses. The Maternal and Child Health Unit contributed to the standardization of pediatric vaccination pathways and operational procedures, while the Primary Care Unit and Primary Care Pediatricians supported territorial coordination, family counselling, identification of children requiring follow-up, and shared reporting with vaccination centers. The Pharmaceutical Unit ensured vaccine procurement, distribution, stock monitoring, and supply continuity across districts. The Information Systems Unit supported data extraction, integration of demographic and vaccination data, Sinfonia updates, dashboard development, and data-quality checks. The Health Directorate provided strategic supervision and alignment with public health priorities, while the Administrative Directorate supported organizational coordination, human-resource planning, and logistical arrangements.

### 2.2 Study timeline

The study timeline was structured into three main periods. The 2022 P01C coverage value was used as the historical reference level, representing performance before the observed decline. The 2023 data were used as the pre-intervention baseline to identify the reduction in vaccination coverage and analyze the organizational, informational, and logistical factors associated with the decline. The improvement plan was implemented progressively during 2024, and the 2024 annual data were used for the post-intervention assessment.

Outcome evaluation was based on the descriptive comparison of annual P01C vaccination coverage between 2023 and 2024, while 2022 was retained as a historical reference to assess whether the post-intervention value returned to the pre-decline level. Intermediate process indicators were compared between 2023 and 2024 to describe the organizational mechanisms accompanying the observed change.

### 2.3 Definition and operationalization of the M.A.S.T.E.R. methodology

M.A.S.T.E.R. was used as a structured QI framework. It is not a clinical intervention, a statistical model, or a measurement scale, but an operational framework designed to organize established QI methods into a sequential and iterative pathway. The framework was not developed specifically for this study. Previous healthcare applications have used M.A.S.T.E.R. to combine process mapping, root cause analysis, prioritization of corrective actions, iterative implementation, performance monitoring, and sustainability planning. These applications support the feasibility of the approach in healthcare settings, but do not constitute formal validation of M.A.S.T.E.R. as a standardized or universally validated instrument.

In this study, M.A.S.T.E.R. was adapted to the pediatric vaccination process and operationalized through six phases. Mapping & Contextualization defined the process boundaries, stakeholders, inputs, activities, outputs, and recipients of the vaccination process. Analysis & Diagnosis identified the organizational, informational, and logistical factors associated with the decline in P01C coverage. Strategic Design translated the identified critical issues into prioritized corrective actions. Testing & Adaptive Execution introduced the selected interventions progressively through PDSA cycles. Embedding & Sustainability incorporated improvement actions into routine procedures, monitoring systems, and clearly assigned responsibilities. Resilience & Renewal defined early-warning indicators and corrective mechanisms to support prospective monitoring and reduce the risk of future declines in vaccination coverage.

The operational tools used within the M.A.S.T.E.R. phases were SIPOC for process mapping, the 5 Whys technique for root cause analysis, an impact-feasibility matrix for prioritization, PDSA cycles for iterative testing, a sustainability checklist for standardization and accountability, and a risk model for prospective monitoring. Therefore, the reproducibility of the approach depends not on M.A.S.T.E.R. as a proprietary or stand-alone instrument, but on the explicit sequence of phases, tools, data sources, responsibilities, and monitoring procedures described in the present study.

### 2.4 Mapping & Contextualization

The first phase was devoted to understanding the organizational context, defining the vaccination process, and identifying the stakeholders involved. During this phase, the main problem (a decline in P01C vaccination coverage in 2023) was defined, and the project objective was clarified: to restore coverage to the level recorded in 2022 and improve the reliability, timeliness, and efficiency of the pediatric vaccination process.

To describe the process in a systematic way, the SIPOC diagram was used to identify suppliers, inputs, key activities, outputs, and recipients of the vaccination process. The SIPOC analysis also clarified the contribution of each organizational actor to the vaccination pathway, distinguishing surveillance, delivery, data management, logistics, clinical-territorial coordination, and strategic governance functions. This tool helped define the boundaries of the process and highlight the interdependencies among vaccination centers, Primary Care Pediatricians, information systems, pharmaceutical logistics, administrative functions, and strategic governance.

### 2.5 Analysis & Diagnosis

The second phase was devoted to analyzing the available data and identifying the factors that had contributed to the decline in vaccination coverage. Data on 24-month-old children were extracted from the Sinfonia platform and integrated with demographic databases and data streams from Pediatric Vaccination Centers.

Because P01C is a nationally defined indicator, its calculation followed the official technical specifications used for national monitoring rather than a locally defined formula. The P01C indicator was calculated according to the protocol of the National Vaccine Registry, in accordance with Law No. 119/2017 and the Ministerial Decree of September 17, 2018. The indicator was calculated as:


$$P01C=\frac{\begin{array}{l} \text{Number of individuals}\;\mathrm{up}\;\text{to}\;24\;\text{months of}\;\mathrm{age}\;\\ \mathrm{who}\;\text{have received}\;a\;\text{full course of}\;\\ \text{vaccination}\;(3\;\text{doses}) \end{array}}{\begin{array}{l} \text{Number of individuals in the}\;\\ \text{respective birth cohort}\; \end{array}}*100$$

The numerator includes:

- vaccinations administered in the region of residence, recorded via the VSX data stream;
- vaccinations administered in other regions to residents, recorded via the VSM data stream;
- vaccinations not administered in the region, excluding cases of prior natural immunity, recorded via the VNX data stream, reason “08”.

The denominator refers to the regional birth cohort, excluding individuals who could not be located, as identified through the VNX data stream, reason “04”.

The data are updated through the Regional Vaccination Registries, ensuring complete coverage of the resident and domiciled population and supporting the continuous monitoring of vaccination campaigns. To investigate the causes of the observed decline, the 5 Whys technique was used, making it possible to link the reduction in the indicator to operational, informational, and organizational issues present at different points in the vaccination process.

### 2.6 Strategic Design

The third phase was dedicated to designing improvement measures. Based on the critical issues identified during the diagnostic phase, corrective actions were selected and prioritized using an impact-feasibility matrix. The matrix made it possible to distinguish priority interventions, characterized by high expected impact and greater feasibility in the short term, from more complex actions to be planned in a subsequent phase.

Particular attention was paid to standardizing active outreach, improving data quality, training operators, developing a monitoring dashboard, strengthening coordination with local health authorities, and reviewing vaccination logistics.

### 2.7 Testing & Adaptive Execution

The fourth phase involved the operational implementation of priority interventions. Implementation was managed as an adaptive process using the PDSA cycle, aimed at planning interventions, testing them progressively, monitoring their effects, and making any necessary adjustments.

The interventions were introduced progressively across the districts of ASL Napoli 3 Sud and included the standardization of active outreach, the use of a digital calendar, the management dashboard, the strengthening of protocols with Primary Care Pediatricians, data integration, review of the vaccine distribution process, staff training, and temporary reinforcement of personnel in districts with the highest workload.

These interventions were implemented as a coordinated multi-component programme rather than as separate sequential interventions.

### 2.8 Embedding & Sustainability

The fifth phase aimed to integrate these measures into routine operational practice. To this end, a checklist was used to define operational standards, the frequency of checks, and the responsibilities of the units involved.

The plan included monthly monitoring of indicator P01C, cross-district comparisons, periodic reviews of operating procedures, audits of recalls and reporting, collection of user feedback, and periodic training sessions for Pediatric Vaccination Center and Primary Care Pediatrician operators.

### 2.9 Resilience & Renewal

The sixth phase introduced a risk model designed to identify potential deviations in the P01C indicator at an early stage. The model considered indicators related to vaccination coverage, Sinfonia registration timeliness, active outreach, vaccination logistics, and operational overload across districts, linking each monitored area to potential corrective actions.

### 2.10 Process indicators and data sources

Intermediate process indicators were selected to describe the main organizational steps targeted by the multi-component improvement programme and to support implementation monitoring. For each indicator, the same operational definition was applied to 2023 and 2024 whenever possible. However, because data cleansing, dashboard development, digital scheduling, and standardized reporting were themselves part of the improvement programme, changes in data recording completeness, traceability, and timeliness may have contributed to some of the observed differences between years.

Recalls carried out were defined as documented outbound contacts made by Pediatric Vaccination Centers to families of eligible children who were not recorded as up to date or required follow-up. They were measured as the number of contacts recorded in the digital calendar, Pediatric Vaccination Center logs, and outbound call reports. The same definition was applied to both years, although the introduction of more standardized recall procedures and digital recording in 2024 may have improved the completeness of recall documentation.

Rescheduled and completed appointments were defined as appointments rebooked after no-shows, postponements, or vaccination delays and subsequently completed with vaccine administration. They were measured using digital vaccination schedules and Pediatric Vaccination Center records. The same event definition was used for both years, but the introduction of the digital calendar may have improved the traceability of rescheduled appointments in 2024.

Late registrations in Sinfonia were defined as vaccination records entered into the Sinfonia platform more than 7 days after vaccine administration. This indicator was measured using the interval between the administration date and the registration date recorded in Sinfonia and in Epidemiology and Prevention Unit–Information Systems control reports. Because this indicator directly reflects data timeliness, observed improvements may represent both faster operational recording and better monitoring of delayed entries.

Adherence by Primary Care Pediatricians to shared protocols was defined as participation in the agreed Pediatric Vaccination Center–Primary Care Pediatrician reporting and coordination pathway, including regular reporting and attendance or contribution to operational coordination activities. It was measured using Primary Care Pediatrician reports, Primary Care Unit/Pediatric Vaccination Center audit records, and reports from operational meetings. The same criteria were applied in both years, although the formalization of protocols in 2024 may have improved documentation of Primary Care Pediatrician participation.

Vaccine distribution time was defined as the time interval between vaccine request and delivery to the requesting district or vaccination service. It was measured using Pharmaceutical Unit logistics flows, district requests, and delivery records. The same measurement approach was used in both years, based on request and delivery dates available in the logistics documentation.

Districts with the highest recovery rates were identified as the districts that were initially below the local health authority average and showed the largest increase in P01C coverage during the post-intervention assessment year. This indicator was measured using the management dashboard and cross-district P01C monitoring reports. Because the dashboard improved the visibility and comparability of district-level data in 2024, this indicator should be interpreted as descriptive of inter-district variation rather than as evidence of district-specific intervention effectiveness.

### 2.11 Statistical analysis

A descriptive statistical analysis was performed to summarize annual P01C vaccination coverage and intermediate process indicators. Annual P01C coverage values were available to the study team as aggregate percentages derived from the official monitoring system. The corresponding annual numerator and denominator counts were not available in the analytical dataset used for this quality improvement evaluation. Therefore, P01C coverage was reported as annual percentages only. Absolute changes were expressed as percentage-point differences, while relative changes were calculated as percentage change compared with the previous year. Intermediate process indicators were summarized descriptively using counts, percentages, mean values, absolute changes, and relative changes, according to the type of indicator. For indicators expressed as percentages, absolute changes were reported as percentage-point differences, while relative changes were calculated as percentage change from 2023 to 2024. No confidence intervals, *p*-values, or statistical tests for differences between proportions were calculated because the underlying individual-level records and annual numerator and denominator counts for P01C were not available. No interrupted time-series analysis was performed because the available data consisted of three annual aggregate observations, from 2022 to 2024, and did not include a sufficiently long sequence of repeated pre- and post-intervention measurements. No multivariable statistical modelling or adjustment for potential confounders was performed.

### 2.12 Ethics statement

This study was conducted as an organizational quality improvement and public health monitoring initiative within the pediatric vaccination process of ASL Napoli 3 Sud. The analysis was based on aggregated, routinely collected administrative and organizational data related to vaccination coverage, process indicators, and service performance. No individual-level identifiable data were used, and no direct intervention on patients or involvement of children, families, or healthcare professionals as research participants was performed.

Therefore, according to applicable institutional procedures, formal ethics committee approval and individual informed consent were not required. All activities were conducted in accordance with principles of confidentiality, data protection, and responsible use of health-related information.

## 3 Results

Following the study timeline, the 2022 P01C value was interpreted as the historical reference level, 2023 as the pre-intervention baseline year, and 2024 as the implementation and post-intervention assessment year. The implementation of a multi-component improvement programme, structured through the M.A.S.T.E.R. framework, was temporally associated with a recovery in vaccination coverage among 24-month-old children. After dropping to 92.75% in 2023, coverage rose to 94.57% in 2024, returning to the historical pre-decline level recorded in 2022, although remaining below the national benchmark of greater than 95%. This corresponded to an absolute increase of 1.82 percentage points and a relative increase of 1.96% compared with 2023. This pattern indicates restoration of previous performance rather than improvement beyond the historical reference level.

The post-intervention period was accompanied by changes in multiple aspects of the vaccination process, particularly active outreach, digital scheduling, data integration, Pediatric Vaccination Center–Primary Care Pediatrician protocols, vaccination logistics, staff training, and periodic monitoring. Intermediate indicators were consistent with operational changes in the process: the number of documented follow-up contacts increased from 1,248 in 2023 to 2,136 in 2024; rescheduled and completed appointments rose from 286 to 612; late entries into Sinfonia, defined as records entered more than 7 days after administration, decreased from 21.4 to 8.6%, with the average time to entry decreasing from 9.8 to 3.6 days; adherence of Primary Care Pediatricians to shared protocols increased from 64/118 (54.2%) to 103/118 (87.3%); and the average time from vaccine request to delivery decreased from 7.4 to 3.1 days. The three districts that were initially most critical showed the largest increases in P01C coverage, amounting to +3.4, +3.1, and +2.8 percentage points, respectively.

### 3.1 Recovery in P01C vaccination coverage

An analysis of indicator P01C showed a decline in vaccination coverage in 2023 and a subsequent recovery in 2024. Coverage decreased from 94.57% in 2022 to 92.75% in 2023, before returning to 94.57% in 2024 during the post-intervention assessment period. Compared with 2023, this represented an absolute increase of +1.82 percentage points and a relative increase of +1.96%. However, the 2024 value matched the historical pre-decline level recorded in 2022 and remained below the national benchmark of greater than 95% (see Table 1).

**Table 1:** Annual P01C vaccination coverage and descriptive changes, 2022–2024.

Year	Vaccination coverage P01C	Absolute change vs previous year	Relative change vs previous year
2022	94.57%	-	-
2023	92.75%	−1.82 percentage points	−1.92%
2024	94.57%	+1.82 percentage points	+1.96%

P01C coverage values were available as aggregate annual percentages from the official monitoring system. Annual numerator and denominator counts were not available in the analytical dataset used for this quality improvement evaluation and therefore could not be reported.

P01C coverage decreased from 94.57% in 2022 to 92.75% in 2023, corresponding to an absolute change of −1.82 percentage points and a relative change of −1.92%. During the post-intervention assessment year, coverage returned to 94.57% in 2024, corresponding to an absolute change of +1.82 percentage points and a relative change of +1.96% compared with 2023. Therefore, the 2024 value should be interpreted as a restoration of the 2022 coverage level and as a partial realignment of vaccination performance to the pre-decline level, rather than as an improvement beyond historical performance or beyond the national benchmark.

These values describe the observed annual variation in coverage.

#### 3.1.1 Intermediate process indicators

Intermediate process indicators were analyzed to describe the organizational changes observed during the implementation of the multi-component improvement programme. These indicators referred to active outreach, appointment follow-up, timeliness of registration in Sinfonia, involvement of Primary Care Pediatricians, vaccination logistics, and inter-district variability. The objective of this analysis was to document the final coverage change together with the main organizational processes targeted by the M.A.S.T.E.R.-structured pathway and to support interpretation of the mechanisms accompanying the observed recovery in P01C coverage.

The intermediate indicators were calculated by comparing 2023, corresponding to the pre-intervention baseline year, with 2024, corresponding to the implementation and post-intervention assessment year. Their operational definitions, data sources, and measurement methods are described in Section 2.10. Data sources included the Sinfonia platform, digital vaccination schedules, Pediatric Vaccination Center records, reports from the Epidemiology and Prevention Unit, Primary Care Pediatrician reports, Pharmaceutical Unit logistics flows, and management dashboards. The same operational definitions were applied to both years whenever possible. However, because digital scheduling, dashboards, data cleansing, and standardized reporting were part of the improvement programme, some observed changes may partly reflect improved data capture, traceability, or recording timeliness rather than only changes in underlying operational performance.

Table 2 presents the baseline and endline values of the main intermediate process indicators used to monitor implementation of the multi-component improvement programme between 2023 and 2024.

**Table 2:** Intermediate process indicators before and after implementation of the multi-component improvement programme, 2023–2024.

Process indicator	Unit of measurement	2023 baseline	2024 endline	Absolute change	Relative change
Documented recalls carried out	n	1,248	2,136	+888	+71.2%
Rescheduled and completed appointments	n	286	612	+326	+114.0%
Late sinfonia registrations (>7 days after administration)	%	21.4	8.6	−12.8 pp	−59.8%
Mean registration delay in Sinfonia	days	9.8	3.6	−6.2	−63.3%
PLS adherence to shared protocols	%	54.2	87.3	+33.1 pp	+61.1%
Regular monthly PLS reporting	%	47.5	82.2	+34.7 pp	+73.1%
Mean vaccine request-to-delivery time	days	7.4	3.1	−4.3	−58.1%
Vaccine orders processed within 72 h	%	58.2	89.6	+31.4 pp	+54.0%

PLS, primary care pediatricians; pp, percentage points. Relative change was calculated as: (2024 value − 2023 value)/2023 value × 100. For indicators expressed as percentages, both absolute percentage-point changes and relative percentage changes are reported. Data sources included digital vaccination schedules, Pediatric Vaccination Center records, Sinfonia data, Epidemiology and Prevention Unit–Information Systems control reports, Primary Care Pediatrician reports, Pharmaceutical Unit logistics flows, district requests, delivery records, and management dashboards.

Table 2 summarizes the intermediate process indicators used to monitor implementation of the multi-component improvement programme. Compared with 2023, documented recalls increased by 71.2%, rescheduled and completed appointments increased by 114.0%, late Sinfonia registrations decreased by 12.8 percentage points, and mean registration delay decreased by 63.3%. Adherence of Primary Care Pediatricians to shared protocols increased from 54.2 to 87.3%, while regular monthly reporting increased from 47.5 to 82.2%. Vaccine logistics also changed descriptively, with mean request-to-delivery time decreasing from 7.4 to 3.1 days and the proportion of orders processed within 72 h increasing from 58.2 to 89.6%.

The three districts that were initially most critical and below the local health authority average showed the largest P01C recoveries during the post-intervention assessment year, corresponding to +3.4, +3.1, and +2.8 percentage points, respectively. This finding was used descriptively to monitor inter-district variability and should not be interpreted as evidence of district-specific intervention effectiveness.

Overall, the intermediate indicators showed changes consistent with the organizational processes targeted by the improvement programme. The increase in documented recalls and rescheduled appointments suggests greater traceability of active outreach and appointment recovery; the reduction in late Sinfonia registrations reflects improved timeliness of data entry; the increase in Primary Care Pediatrician adherence indicates stronger documentation of territorial coordination; and the reduction in vaccine distribution times is consistent with improved logistics monitoring. These indicators support implementation monitoring and contextual interpretation of the observed recovery.

### 3.2 Results of the Mapping & Contextualization phase

The Mapping & Contextualization phase enabled the pediatric vaccination process to be described as an integrated system rather than a sequence of isolated activities. The SIPOC (Table 3) diagram clarified the roles of the various stakeholders, the necessary inputs, the operational activities, the expected outputs, and the final recipients of the process.

**Table 3:** SIPOC diagram of the pediatric vaccination process for indicator P01C.

Supplier	Input	Process	Output	Customer
UOC SEP/Epidemiology and Prevention Unit	Planning and monitoring	Launch of planning and policy-making initiatives	Monitoring	24-month-olds and their families
Pharmaceutical Unit/UOFI	Vaccines and medical supplies	Vaccine procurement, stock management, and distribution.	Vaccine availability and supply continuity	Pediatric Vaccination Centers and districts
Primary Care Pediatricians	Information about families	Vaccination coordination and reporting	Timely and up-to-date reports	Pediatric vaccination centers
Maternal and Child Health Unit	Vaccination guidelines and protocols	CVP demand planning and operational coordination	Consistency and quality of the vaccination service	CVP and districts
Health Directorate	Strategic direction and clinical supervision	Coordination of the Maternal and Child Health Unit and overall supervision	Strategic management of the vaccination programme	Maternal and Child Health Unit, CVP, Local Health Authority
Primary Care Unit	Institutional communications and organisational information	Management and involvement of community health workers in the vaccination campaign	Increased uptake and participation	Families, PLS, CVP
Information Systems Unit	Software and IT systems	Data recording and monitoring	Reports and alerts for monitoring	Administrative Management and staff
Administration Directorate	Human resources and logistics organisation	Overall supervision and coordination	Optimised process	Local community and stakeholders

The use of the SIPOC model revealed that the performance of indicator P01C depended on the synchronization of several organizational components: identification of the target population, appointment scheduling, administration, data recording, reporting, vaccine logistics, and managerial monitoring.

### 3.3 Results of the Analysis & Diagnosis phase

The Analysis & Diagnosis phase identified the main organizational and informational causes associated with the decline in vaccination coverage in 2023. The use of the 5 Whys technique (Table 4) revealed critical issues throughout the entire process, with particular reference to active outreach, birth tracking, data quality, coordination with Primary Care Pediatricians, vaccination logistics, and operational variability across districts.

**Table 4:** Summary of critical issues identified using the 5 Whys.

Area/Unit	Main issue	Scope of intervention
UOC SEP	Room for improvement in data reporting	Dashboards and regular reporting
CVP	Non-automated tracking of births	Integrated birth-vaccination system
CVP	Non-standardised active outreach	Standardisation of follow-up procedures
CVP	Insufficient staff for follow-up	Temporary increase in staffing levels
Primary Care Unit/PLS	Irregular communications and lack of shared indicators	Shared protocols and audits
PLS	Delays in updating data on Sinfonia	Strengthening of CVP-PLS coordination
Maternal and Child Health Unit	Delays and operational inconsistencies	Standardisation of procedures
UOFI Pharmaceuticals	Delays in vaccine distribution	Review of the distribution process
Information Systems Unit	Mismatch between demographic and vaccination data	Data integration and cleansing

The analysis showed that the decline in the indicator was not attributable to a single cause, but to a set of interdependent factors. Among these, the lack of standardization in recall procedures, delays in updating data on Sinfonia, incomplete integration between demographic and vaccination data, operational variability across districts, and challenges in vaccine distribution were particularly significant.

### 3.4 Results of the Strategic Design phase

The Strategic Design phase transformed the identified critical issues into a structured action plan. The impact-feasibility matrix (Table 5) enabled the selection of priority actions, prioritizing those with a high expected impact and greater feasibility in the short term.

**Table 5:** Prioritisation of actions using the impact-feasibility matrix.

Area of activity	Planned action	Expected impact	Feasibility
Active case finding	Standardisation of recalls using multi-channel reminders and a digital calendar	High	High
Monitoring	Management dashboard with alerts for deviations	High	High
Cohort tracking	Integrated birth-vaccination system	High	Medium
CVP-PLS coordination	Shared protocols, standardized reporting and periodic audits	High	Medium
Vaccination logistics	Review of the distribution flow and district safety stocks	Medium-high	Medium
Data quality	Data cleansing and integration via the SEP-Information Systems task force	High	High
Operational expertise	Ongoing training for CVP and PLS staff	Medium-high	High
Human resources	Temporary reinforcement in districts with a higher workload	Medium-high	Medium

The priority actions identified were the standardization of active outreach calls, the introduction of a digital calendar, data cleansing and integration, the management dashboard, and operator training. Actions involving greater organizational complexity, such as the integration of birth and vaccination records, the strengthening of protocols with Primary Care Pediatricians, and the review of logistics, were planned as phased but strategic interventions to support the sustainability of the results.

### 3.5 Results of the Testing & Adaptive Execution phase

The Testing & Adaptive Execution phase enabled the transition from design to operational implementation of the interventions. The use of the PDSA cycle (Table 6) allowed for the gradual introduction of actions in the districts, monitoring of their effects, and adaptation of procedures based on operator feedback and the trend of indicator P01C.

**Table 6:** Application of the PDSA cycle in the Testing & Adaptive Execution phase.

Phase PDSA	Application within the project
Plan	Identification of priority measures: standardised recall system, digital calendar, management dashboard, PLS protocols, data integration and logistical review
Do	Gradual implementation of the measures across the districts of ASL Napoli 3 Sud
Study	Monitoring of indicator P01C, comparison between districts and collection of feedback from staff
Act	Resolution of remaining issues and adaptation of operational procedures

During this phase, the following were introduced: standardized recall procedures, multi-channel reminders, a digital calendar, a management dashboard, reporting protocols with Primary Care Pediatricians, operator training, a review of the distribution workflow, and temporary staff reinforcement in districts with the highest operational workload.

The PDSA cycle facilitated a flexible application of interventions, avoiding a rigid implementation model and allowing for progressive adjustments based on identified critical issues.

### 3.6 Results of the Embedding & Sustainability phase

The Embedding & Sustainability phase allowed for the consolidation of interventions and their transformation into organizational routines. The sustainability checklist (Table 7) defined the elements to be monitored, the frequency of checks, and the responsible personnel for maintaining standards.

**Table 7:** Sustainability checklist.

Items to be checked	Frequency	Responsible unit/personnel
Monitoring of P01C and cross-district comparison	Monthly	SEP/CVP Clinical Unit
Review of operating procedures	Half-yearly	Maternal and Child Health Clinical Unit/Primary Care Clinical Unit
Audit of recalls, reporting and data updates	Periodic	CVP/PLS/Primary Care Clinical Unit
User feedback	Continuous	CVP/Information Systems Clinical Unit
Training and refresher courses for staff	Periodic	Maternal and Child Health Clinical Unit/CVP

This phase strengthened the continuity of monitoring, the accountability of the units involved, and the integration of interventions into the routine organizational management system. The maintenance of monthly reports, cross-district comparisons, periodic audits, and ongoing training was intended to support the stability and sustainability of the P01C recovery over time.

### 3.7 Results of the Resilience & Renewal phase

The Resilience & Renewal phase oriented the vaccination system toward a proactive approach to preventing new deviations. The risk model (Table 8) made it possible to identify early warning signs of critical issues and to associate each sign with a potential renewal or improvement action.

**Table 8:** Risk model for the Resilience & Renewal phase.

Monitored area	Risk indicator	Renewal/improvement initiative
P01C coverage	Shortfall compared to the expected figure	New targeted recall cycle
Data recording	Delays or incomplete data in Sinfonia	Review of information flows
Active outreach	Failure to contact eligible individuals	Strengthening of the digital calendar
Vaccination logistics	Delays in distribution or stock issues	Review of the distribution process
District organisation	Operational overload or management inconsistencies	Organisational support and annual audit

The risk model made it possible to link the maintenance of vaccination coverage not only to the monitoring of the final indicator but also to the tracking of organizational factors that may foreshadow a decline in coverage. The combination of annual audits, dashboards, district monitoring, and renewal initiatives was intended to strengthen the system’s ability to identify emerging critical issues, adapt to new organizational challenges, and generate operational learning that may be tested in other areas of prevention.

### 3.8 Overall summary of results

Overall, the M.A.S.T.E.R. methodology was used as an organizational framework to structure and coordinate a multi-component response to the decline in the P01C indicator and to guide the redesign of the vaccination process. The recovery in coverage from 92.75% in 2023 to 94.57% in 2024 restored the value observed in 2022, but did not exceed the historical pre-decline level.

This recovery occurred alongside documented changes in intermediate indicators related to recalls, appointment recovery, timeliness of registration, Primary Care Pediatrician adherence, vaccine distribution, and inter-district variability. Taken together, the results suggest that the integration of data analysis, operational standardization, and territorial coordination may have contributed to the realignment of organizational performance.

## 4 Discussion

This study describes the use of the M.A.S.T.E.R. methodology as an organizational framework to coordinate a multi-component improvement programme addressing the decline in the P01C indicator within the pediatric vaccination process of ASL Napoli 3 Sud. P01C coverage increased from 92.75% in 2023 to 94.57% in 2024, returning to the level recorded in 2022. Although this recovery is operationally relevant for a population-level prevention indicator, the 2024 value remained below the national benchmark of greater than 95% (24). Therefore, the result should be interpreted as restoration of previous performance rather than improvement beyond the historical level or full achievement of the national target.

Because of the before-and-after QI design, the absence of a concurrent control group, and the simultaneous implementation of multiple activities, the observed recovery should be interpreted as a post-implementation change rather than as evidence of causal effectiveness. The descriptive nature of the analysis also limited the possibility of estimating the independent contribution of the M.A.S.T.E.R. framework or of individual programme components. Several alternative explanations should also be considered. The increase in P01C coverage may have been influenced by recovery of routine preventive services after the COVID-19 pandemic, normal year-to-year variation, seasonal or within-year patterns not captured by annual data, changes in staffing or workload distribution, increased public or professional awareness of vaccination, communication initiatives, or other concurrent organizational actions. This is particularly relevant because global and national evidence shows that routine childhood vaccination coverage remains vulnerable to disruptions, and that even modest declines may increase the risk of vaccine-preventable disease resurgence (25, 26). The intermediate process indicators suggest that the recovery in P01C coverage occurred alongside changes in several organizational components of the vaccination pathway. Documented follow-up contacts, rescheduled appointments, Sinfonia registration timeliness, Primary Care Pediatrician adherence to shared protocols, vaccine delivery times, and inter-district monitoring all changed in the expected direction. These findings are consistent with the multifactorial nature of the 2023 decline, which involved non-standardized outreach, incomplete integration between demographic and vaccination data, delays in Sinfonia updates, inconsistent coordination between Pediatric Vaccination Centers and Primary Care Pediatricians, operational variability across districts, logistical challenges, and limited staff dedicated to follow-up. However, these process indicators should be interpreted cautiously, because some changes may partly reflect improved documentation, dashboard use, data cleansing, and standardized reporting rather than only changes in underlying operational performance.

The findings are consistent with previous quality improvement studies aimed at increasing vaccination coverage, which suggest that effective interventions are rarely based on a single action. Fu et al. reported improvements in overall and on-time immunization rates after a comprehensive QI programme targeting underserved children (27), while Harder et al. found that QI efforts in pediatric primary care were associated with increased immunization coverage across age groups and vaccine series, with partial validation through population-based registry data (28). Evidence on reminder and recall systems also supports the use of active outreach strategies in vaccination programmes (10), and provider-focused interventions, including reminders, audit and feedback, education, and practice-based QI activities, have been reported to increase child and adolescent vaccination rates (29). However, unlike many stronger evaluations, the present study was not randomized, controlled, or based on interrupted time-series analysis; therefore, the comparison with previous vaccination QI studies supports the plausibility of the approach but not causal attribution in this setting.

The contribution of M.A.S.T.E.R. in this study should be interpreted primarily as methodological and organizational. The framework did not function as a clinical intervention or validated measurement instrument, but as a structured way to combine established QI tools into a single pathway linking diagnosis, prioritization, adaptive execution, routine embedding, sustainability, and resilience. This differs from traditional PDCA/PDSA cycles, which mainly focus on iterative testing, and from Lean Six Sigma or DMAIC approaches, which emphasize waste reduction, variation control, and process optimization. The use of PDSA cycles, DMAIC, Lean tools, A3 thinking, dashboards, and audit mechanisms is consistent with previous healthcare QI literature (30–35). In this study, their integration within the M.A.S.T.E.R. framework helped organize the response to a nationally monitored indicator deviation, but does not constitute formal validation of the framework as universally applicable.

Digital tools represented an enabling component of the improvement pathway. The management dashboard, integration of demographic and vaccination data, and enhanced updating of Sinfonia may have supported more timely identification of critical issues. This is consistent with literature on electronic registries and immunization platforms, which highlights their role in coverage surveillance, reduction of information gaps, and planning of interventions (36). Similarly, the Embedding & Sustainability and Resilience & Renewal phases were intended to support maintenance of the observed recovery through standardized procedures, assigned responsibilities, periodic audits, dashboard-based monitoring, and early-warning indicators. This is consistent with evidence that QI gains may decline if they are not incorporated into routine procedures and control systems, and with the concept of organizational resilience in healthcare (37–39).

The study has several limitations. First, it was conducted in a single local health authority and did not include a concurrent control or comparison group; therefore, causality cannot be established. Second, multiple activities were implemented together, making it impossible to determine which component contributed most to the observed recovery. Third, the study relied on routinely collected administrative and operational data, including vaccination records, Sinfonia data, Pediatric Vaccination Center logs, Primary Care Pediatrician reports, logistics flows, and dashboards; these sources may be affected by incomplete records, delayed data entry, reporting bias, and inconsistencies between demographic and vaccination databases. Fourth, only three annual aggregate observations were available, and annual numerator and denominator counts were not included in the analytical dataset; therefore, inferential analyses and evaluation of pre-existing trends, seasonality, regression to the mean, or natural annual fluctuation were not possible. Fifth, the follow-up was limited to the 2024 post-intervention year, and no formal long-term sustainability assessment was performed. The Hawthorne effect may also have influenced short-term reporting practices, adherence to procedures, or engagement with recall activities.

Finally, wider applicability should be interpreted cautiously. The findings should not be taken as evidence that M.A.S.T.E.R. is directly generalizable to other prevention programmes. Differences in population characteristics, baseline coverage, information systems, staffing, organizational culture, primary care engagement, and regional governance may affect implementation elsewhere. Some organizational elements of the approach—such as structured process analysis, stakeholder coordination, standardized procedures, dashboards, audit cycles, and prospective monitoring—may be relevant to other prevention indicators if adapted to local context and evaluated with stronger designs (40). Future studies should extend follow-up, include formal sustainability assessments, and evaluate whether combined use of reminders, recalls, provider engagement, data-quality improvement, and logistical redesign produces stable effects over time, considering that multifactorial interventions have shown robust evidence in improving immunization coverage among children and adolescents (41).

## 5 Conclusion

The implementation of a multi-component improvement programme organized through the M.A.S.T.E.R. framework was followed by an increase in vaccination coverage among 24-month-old children in the ASL Napoli 3 Sud local health authority, from 92.75% in 2023 to 94.57% in 2024. This value matched the coverage level recorded in 2022, indicating restoration of previous performance rather than improvement beyond the historical pre-decline level.

The study shows the feasibility of using M.A.S.T.E.R. as an organizational framework to structure the response to a decline in a nationally monitored vaccination indicator. The observed recovery occurred alongside changes in active outreach, data timeliness, coordination with Primary Care Pediatricians, vaccination logistics, and inter-district monitoring. The findings suggest that combining outcome indicators with intermediate process indicators may help local health authorities identify actionable bottlenecks in immunization pathways.

M.A.S.T.E.R. should therefore be interpreted not as an isolated intervention, but as a framework for coordinating multiple improvement activities within a specific local health authority. Further evaluations in different organizational settings, using stronger study designs and longer prospective monitoring, are needed to assess transferability, sustainability, and independent contribution.

## Data Availability

The raw data supporting the conclusions of this article will be made available by the authors, without undue reservation.
